# p110α activity at the M-to-G1 transition is critical for cellular proliferation and reentry into the cell cycle

**DOI:** 10.55730/1300-0152.2609

**Published:** 2022-02-08

**Authors:** Onur ÇİZMECİOĞLU

**Affiliations:** Department of Molecular Biology and Genetics, Science Faculty, İhsan Doğramacı Bilkent University, Ankara, Turkey

**Keywords:** Cell cycle, signal transduction, phosphoinositide 3-kinase pathway, temporal regulation, p110α

## Abstract

Phosphoinositide 3-kinase (PI3K) signaling pathway is essential for normal physiology and is impaired in diseases such as premalignant hyperproliferative disorders, primary immunodeficiency, metabolic disorders, and cancer. Although the core PI3K pathway components are known today, a long-standing gap in our knowledge of PI3K signaling concerns how distinct PI3K isoforms and their activity patterns contribute to the functional consequences of pathway upregulation. In order to address this issue, we devised a molecular genetic cell model, which allowed temporal regulation of the indispensable PI3K isoform, p110α in distinct stages of the cell cycle. We found that late M and early G1 presence of p110α is key for proper cell cycle progression, whereas its S-phase abundance was redundant. Our results also emphasize a critical dependence of cell cycle reentry on early G1 activity of p110α. Collectively, our findings provide a temporal perspective to p110α activation and offer insight into which wave of PI3K activity could be essential for cell cycle progression.

## 1. Introduction

The phosphoinositide 3-kinase (PI3K) pathway is often deregulated during carcinogenesis. Class IA PI3Ks are obligate heterodimers, comprised of a catalytic and a regulatory subunit, namely p110 and p85, respectively. PI3Ks receive signals from receptors located at the cell membrane and oncoproteins such as Ras, Fibroblast growth factor receptor, insulin-like growth factor receptor, ERBB2, and G protein-coupled receptors (GPCRs). On the other hand, class IB PI3Ks receive input only from GPCRs (Liu et al., 2009; Guerrero-Zotano et al., 2016; Mayer and Arteaga 2016). *PIK3CA*, *PIK3CB*, and *PIK3CD* genes encode the catalytic class IA isoforms p110α, p110β, and p110δ. They have 5 common domains: an aminoterminal p85binding domain, a RASbinding domain, C2 domain for membrane binding followed by the helical domain, and a carboxyterminal catalytic domain. The regulatory subunit p85 is comprised of 5 different isoforms, namely p85α, p55α, p50α, p85β, and p55γ. These isoforms have 3 mutual regions: interSrc homology 2 (iSH2) domain that is required for p110 binding is flanked by two SH2 domains (Jia et al., 2009; Liu et al., 2009). In the absence of an activatory signal, p85 inherently inhibits catalytic activity of PI3K by binding to the aminoterminus of the p110 subunit via its iSH2 domain (Liu et al., 2009). Adaptor proteins activated by receptor tyrosine kinases, bind to p85 and consequently override p85 mediated inhibition on the p110 subunit. Activated p85–p110 complex in turn, binds phosphatidylinositol 4,5-bisphosphate (PIP_2_) at the cell membrane and converts it to phosphatidylinositol 3,4,5-trisphosphate (PIP_3_) via phosphorylation. Elevated PIP_3_ levels localize Pdk1 and Akt to the cell membrane through interacting with their pleckstrin homology (PH) domain, where Pdk1 induces phosphorylation of Akt at the conserved threonine 308 residue. Akt is then optimally activated when mTOR/Rictor (mTORC2) complex additionally phosphorylates Akt at serine residue 473. Activated Akt then phosphorylates its substrates, such as PRAS40 and TSC2. Phosphorylated TSC2 can no longer mediate its inhibitory effects on mTOR/Raptor (mTORC1) complex. Activated mTORC1, in turn, phosphorylates its targets such as S6K and 4EBP1. Overall, mTORC1 activity results in metabolic activation, protein synthesis, and cellular growth. In addition, phosphorylation of other Akt substrates including Gsk3α, Mdm2, FoxO, p27, and Bad lead to cellular survival and entry into cell cycle (Guertin and Sabatini 2007; Manning and Cantley 2007; Guerrero-Zotano et al., 2016; Mayer and Arteaga 2016).

It has been suggested that PI3K activity govern cell cycle transitions in S, as well as in G2-M phases (Liang and Slingerland 2003). Inhibition of PI3K activity via small molecule inhibitors prohibit entry into the cell cycle (Wymann and Pirola 1998), a process which is directly correlated with PIP_3_ synthesis (Alvarez et al., 2001, 2003). PI3K effectors such as Rac and Cdc42 have been implicated in cyclin D synthesis, and Akt mediated phosphorylation was reported to stabilize c-Myc by blocking its degradation (van Weeren et al., 1998). Catalytic inhibition of PI3K in late-G1 inhibits DNA synthesis and S-phase entry (Jones and Kazlauskas 2001). Besides controlling early stages of the cell cycle, PI3K has been shown to modulate G2 and M phases as well. Inhibiting PI3K in the late-S phase prevents MDCK cells from entering into mitosis, whereas it delays M phase entry in HeLa and NIH3T3 models. (Shtivelman et al., 2002). FoxO and FoxM dependent expression of cyclin B and Plk1 is essential for progression through G2 and PI3K activity can regulate subcellular localization of these transcription factors to determine the timing of mitotic onset (Alvarez et al., 2001; Laoukili et al., 2005).

These observations support the notion that a fine-tuned regulation of PI3K activity is key for proper cell cycle progression. Nevertheless, isoform-specific requirements of PI3K during cell cycle transitions have not been sufficiently characterized. In order to address isoform-specific contributions of PI3K to cell cycle progression, we devised a molecular genetic system allowing expression of singular PI3K isoforms in a p110α and p110β double knock-out (DKO) background (Cizmecioglu et al., 2016). This model alleviates the background PI3K signaling and generates a genetic setting where cellular proliferation is entirely dependent on the expression of the ectopic PI3K isoform. Using this strategy, we analyzed cellular proliferation, Akt activity, and cell cycle kinetics, upon expression of p110α in specific stages of the cell cycle. This was achieved via fusing p110α to domains of proteins that exhibit phase-specific stability in the cell cycle (Geminin and Cdt1) (Sakaue-Sawano et al., 2008). We found that the activity of p110α in late M and early G1 is critical for cellular reproduction and cell cycle reentry. In addition, we determined that the ensuing S phase activation of p110α is rather dispensable for the cell cycle and proliferation. Collectively, our results highlight the importance of the early wave of p110α activity in cellular growth and have potential implications concerning the optimum methods for suppressing PI3K activity.

## 2. Materials and methods

### 2.1. Compounds and reagents

Nocodazole, thymidine, and crystal violet were purchased from Sigma. CellTiter-Glo luminescent cell viability kit was obtained from Promega.

### 2.2. Cell culture

p110α flox/flox; p110β flox/flox mouse embryonic fibroblasts (MEFs) and HEK293 cells were grown in Dulbecco’s modified Eagle’s medium (DMEM, high-glucose, L-glutamine and 110 mg/L sodium pyruvate) supplemented with 8% FBS and pen-strep solution (100 IU/mL and 100 mg/mL respectively, Gibco) in a humidified incubator at 37°C. All cell lines used were tested negative for mycoplasma. Fetal bovine serum (FBS) was purchased from Gemini-Bio. MEFs were arrested in prometaphase for 15 h with 50 ng/mL nocodazole (Sigma). Mitotic cells were then removed from the block by shaking them off, washing them three times, and reincubating them in a fresh medium. Thymidine block was conducted by treating the cells with 4 mM thymidine for 19 h. Subsequently, cells were released for 12 h and again blocked with 4 mM of thymidine for another 15 h. MEFs were prepared from embryos at embryonic day 13.5 from p110αflox/flox and p110βflox/flox homozygotes. Primary MEFs were immortalized using the standard 3T3 protocol, and polyclonal knock-outs were generated as described previously (Cizmecioglu et al., 2016)p110α and p110β.

### 2.3. Plasmids and retroviral transductions

Human p110α-Cdt1 and p110α-Geminin degron plasmids were cloned by exchanging human PIK3CA sequence for SV40ST in the pBabe-puro-SV40-Cdt1/Geminin plasmids. These plasmids were a kind gift from David Livingston (Naetar et al., 2014). Retroviral transductions were performed to generate stable MEF lines. Retroviral particles were generated by transfecting HEK293 cells with gag-pol, vsv-g, and degron tagged PIK3CA using lipofectamine 2000 (Invitrogen). At 48 h and 72 h after transfection, supernatants containing retroviral particles were collected, pooled, sterile filtered, and used right away.

### 2.4. Antibodies and Western blotting

Anti-HA, anti-p110α, anti-p110β, anti-p-Akt (S473), anti-p-Akt (T308), anti-p-S6 (S240/244), anti-cyclin B, and anti-cyclin A antibodies were obtained from Cell Signaling Technology (CST). Anti β-actin antibodies were purchased from Sigma. Fluorescent secondary antibodies were obtained from Li-cor. A standard protocol for western blotting was used (Cizmecioglu et al., 2012). In brief, cells were scraped into ice-cold PBS and then lysed with RIPA buffer (Westnet) supplemented with protease/phosphatase inhibitor tablets (Roche), 1 mM sodium orthovanadate (CST), and 1 mM dithiothrietol (Bio-Rad) at 4 °C. A total of 15–20 μg total protein was electrophorased with 10%–12% SDS-PAGE and blotted to nitrocellulose membranes (Bio-Rad). After blocking with 5% fat-free milk in TBS for 30 min, membrane stripes were incubated with primary antibodies in TBS, 5% milk, and 0.1% Tween-20 o/n at 4 °C. After that, the membranes were treated for 2 h at ambient temperature with secondary antibodies diluted in 5% milk-TBS. Signals were detected with a Licor Odyssey CLx device.

### 2.5. Cellular proliferation assays

Crystal violet works as an intercalating dye and allows the quantification of DNA, which is proportional to the number of cells. Crystal violet assays were performed by seeding 2×10^4^ MEF cells in 6-well plates (Corning). Cells were rinsed with PBS and fixed with 10% acetic acid (Sigma), 10% ethyl alcohol for 4 h at room temperature. Staining was performed with 0.2% crystal violet (Sigma), 10% ethyl alcohol for 30 min, followed by a rinse with distilled water, and then the wells were air-dried. Cell-associated crystal violet stain was extracted with 2 mL/well of 10% acetic acid for 20 min on a vertical rocker, and spectrometric measurements were done at an OD of 595 nm. Values were normalized for day 0 in each experiment. As an alternative cellular proliferation assay, Celltiter-Glo (Promega) cellular viability assays were performed. This is a method to determine the number of viable cells based on quantitation of cellular ATP, which is proportional to the number of metabolically active cells. Cells were seeded in 96-well plates at a density of 1,000 cells/well. Viable cells with an active metabolism were assessed after 6 days of seeding by Celltiter-Glo (Promega) at an OD of 560nm. Figures depicted are the average of three independent experiments.

### 2.6. Cell cycle analysis

2×10^5^ cells were centrifuged and treated with RNase (50 mM Tris-HCl, pH 7.5, 10 mM MgCl_2_, 10 mg RNase A/mL; Roche) for 30 min at 37 °C to degrade cellular RNA degraded by RNase treatment. For cytofluorometric measurement of DNA content propidium iodide (30 mg/mL, Sigma), which is a DNA and RNA intercalating dye, was used to stain the cells for 30 min on ice in the dark. FACSSort (Becton Dickinson Biosciences) and the CellQuest software were used to analyze the DNA content of 10^4^ cells.

### 2.7. Statistical analysis

Two-tailed student’s t-test was used for differential comparison between two groups. T-tests were performed in GraphPad Prism. Data are considered significant when p values are < 0.05. All data are presented as mean ± standard deviation unless otherwise stated.

## 3. Results

### 3.1. Generation of an isogenic cell model expressing p110α in distinct stages of the cell cycle

**Figure 1:**
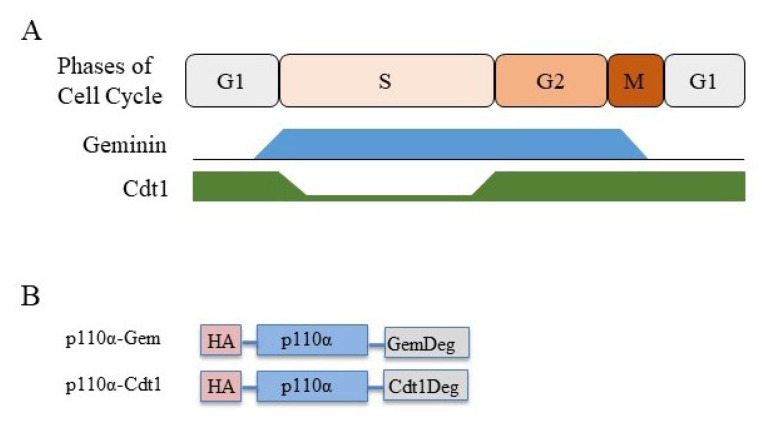
Temporal regulation of protein stability with Geminin and Cdt1 degrons. **A-** A schematic representation of Geminin and Cdt1 protein abundance throughout cell cycle. G1, S, G2 and M represent gap 1, synthesis, gap 2 and mitotic stages of the cell cycle respectively. **B-** A schematic depiction of geminin and Cdt1 tagged p110α constructs. p110α in a pBabe-puro backbone is tagged with an amino terminus HA and a carboxy terminus degron-tag. GemDeg and Cdt1Deg represent Geminin and Cdt1 degrons, respectively.

Our previous work has characterized a molecular genetic system, which enables ablation of class IA PI3K activity in mouse embryonic fibroblasts (MEFs) upon expression of Cre-Recombinase in adenoviral particles (Cizmecioglu et al., 2016). Building upon that model, we generated p110α vectors with C-terminal ‘degron’ sequences (Figure 1A), which facilitate destruction of the tagged protein in certain phases of the cell cycle (Naetar et al., 2014). Notably, geminin degron destabilizes the attached protein in late M and early G1 phases, whereas a Cdt1 degron leads to protein degradation in late G1 and S phases (Figure 1B).

**Figure 2:**
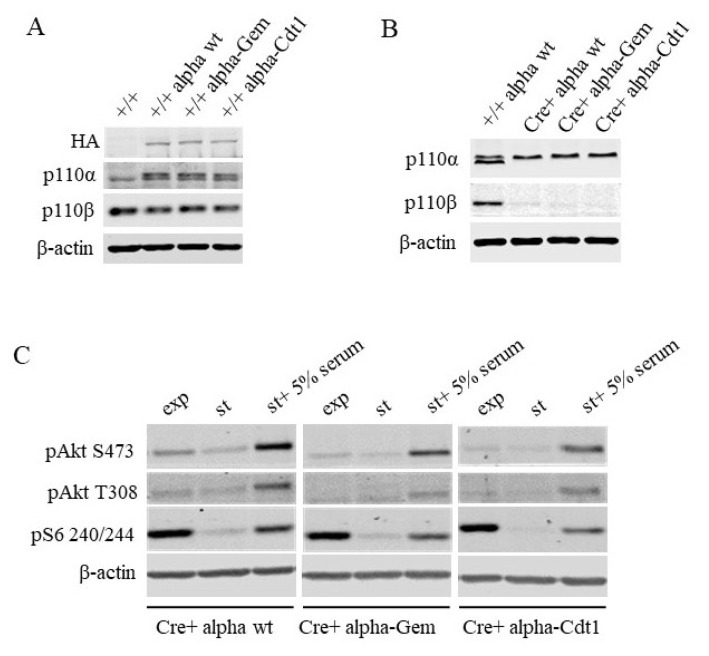
Generation of p110α add-back MEF lines. **A-** MEF lines stably expressing either wild-type (alpha-wt) or Gem/Cdt1 degron tagged p110α. Ectopic protein expression was assessed with anti HA antibodies, whereas anti-p110α and p110β immunoblots determined the level of total p110α and endogenous p110β. **B-** Expression of ectopic p110α constructs upon adenoviral expression of Cre-recombinase. p110α and p110β immunoblots determined the level of ectopic p110α as well as the remaing levels of endogeous p110α and p110β. **C-** DKO+p110α-wt, DKO+p110α-Gem and DKO+ p110α-Cdt1 MEFs were starved and stimulated with 5% FBS. p-Akt (for T308 and S473) and p-S6 (for S240/244) immunoblots were used to measure the effectiveness of PI3K signaling in these cells. Anti β-actin blots serve as loading control for all panels.

We used three viral delivery vectors in our study: p110α-wt, p110α-Geminin Degron (GemDeg; denoted as ‘Gem’ in the remainder of the article), and p110α-Cdt1 Degron (Cdt1Deg; denoted as ‘Cdt1’). These plasmids were retrovirally transduced into our engineered MEFs. Wild type and degron tagged protein expressions were detected at comparable levels in asynchroneously growing cells (Figure 2A). After determining similar levels of protein expression, endogenous *PIK3CA* and *PIK3CB* were knocked out (double knock-out; DKO) of the MEF system with repeated rounds of AdCre infections. Ablation of endogeous p110α and p110β was determined with antibodies specific to p110α and β in immunoblots. Hemagglutinin (HA) epitope and degron-tagged p110α migrates somewhat slower than endogeous p110α. Disappearance of the endogeous p110α signal alongside with p110β is evident upon AdCre infections, depicting effective loxP targeting and knock-out efficacy (Figure 2B). Next, we checked the functionality of our ectopic p110α constructs. Notably, ectopic expression of either degron plasmid is capable of triggering PI3K activation in response to starvation followed by serum stimulation, in a similar manner to the wild type p110α (Figure 2C).

**Figure 3:**
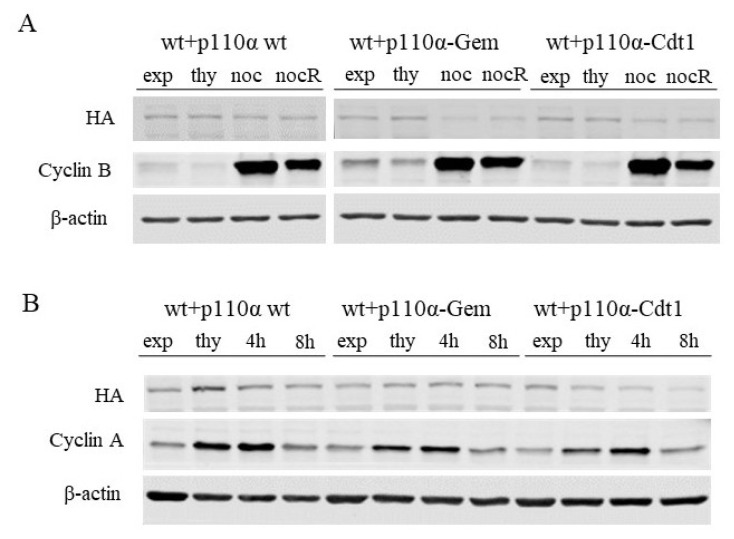
Validation of degron tagged p110α expression in different stages of the cell cycle. **A-** p110α-wt, p110α-Gem and p110α-Cdt1 expressing MEFs were either grown exponentially (exp) or were enriched with thymidine (thy) or nocodozole (noc) treatments. A proportion of noc treated cells were released from the nocodozole block (nocR) and were allowed to progress in the cell cycle for 2 h. Anti HA antibodies detected expression of ectopic p110α, whereas anti cyclin B serves as a marker for M phase. **B-** p110α-wt, p110α- Gem, and p110α-Cdt1 expressing MEFs were either grown exponentially (exp) or were enriched with thymidine (thy) treatment. A proportion of thy treated cells were released from the thymidine block and were released into the cell cycle for indicated time intervals. Anti HA antibodies detected expression of ectopic p110α, whereas anti cyclin A serves as a marker for S phase. Anti β-actin blots serve as loading control for all panels.

After demonstrating that the p110α degron constructs are catalytically active, we set out to determine if these tags allowed destabilization of the conjugated p110α in the desired cell cycle stage. First, we performed nocodozole treatment of p110α-wt, p110α-Gem, and p110α-Cdt1 expressing MEFs. Nocodozole is a reversible microtubule poison, activating the spindle assembly checkpoint and inducing M-phase arrest. Nocodozole arrested cells can be released into M-phase upon washing the drug off with culture media. p110α-wt expression was marginally affected by the nocodozole treatment and subsequent release, implicating the stability of wild type p110α in mitosis. In contrast, p110α-Gem was markedly destabilized at protein level upon nocodozole induced M-phase arrest, an effect that can be partially reversed upon nocodozole washout (Figure 3A, compare lanes 7 and 8). On the other hand, p110α-Cdt1 levels did not seem to respond to a nocodozole block and release (Figure 3A, compare lanes 11 and 12). The reason for observing residual downregulation of ectopic protein levels in nocodozole treated samples is because of reduced cap-dependent translation in mitotically arrested in comparison to exponentially growing cells (Pyronnet et al., 2001). Next, we tried to enrich the cells in S-phase and aimed to determine whether the Cdt1 degron trigger protein destabilization in that phase of the cell cycle. Thymidine is a pyrimidine deoxynucleoside, which is used to synchronize cells in G1/early S phase. We treated our MEFs with thymidine to arrest them in G1-S phase transition and then washed off thymidine and released cells into the cell cycle for indicated periods of time. Release from a thymidine block led to a decrease in the levels of p110α-Cdt1 but not p110α-Gem (Figure 3B, compare lanes 10 with 11–12). Collectively, these results indicate that degron tagged p110α constructs are functional and exhibit cell cycle interval-specific stability.

### 3.2. p110α function at late M-G1-phase is essential for cell proliferation and progression in the cell cycle

**Figure 4:**
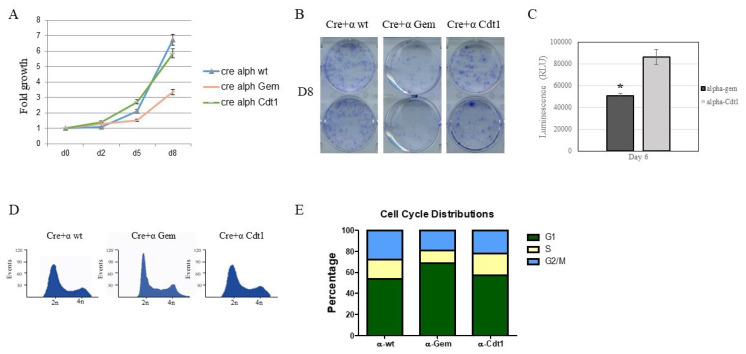
p110α-Cdt1 expressing MEFs exhibit retarded proliferation kinetics in comparison to p110α-Gem. **A-** After 2, 4, 6, and 8 days of growth in 8% FBS-DMEM, cellular proliferation was measured using crystal violet assays (on the left). Error bars indicate standard deviation in 3 independent experiments. **B-** A representative crystal violet assay after 8 days of cell proliferation. **C-** Cell viability of the indicated MEF variants were determined by CellTiter-Glo assay after 6 days of growth. Error bars indicate standard deviation in 3 experiments. * denotes p < 0.05. **D-** p110α-wt, p110α-Gem and p110α-Cdt1 expressing add-back MEFs were analyzed with propidium iodide staining. Histograms of the exponentially growing cells are depicted. **E-** Cell cycle analysis of indicated cells was carried out using the CellQuest software for at least 10^4^ cells.

Next, we checked the proliferative potential of p110α-Gem and p110α-Cdt1 alongside with p110α-wt expressing DKO MEFs. Crystal violet staining of exponentially growing cell populations indicated that p110α-Gem but not p110α-Cdt1 expressing cells exhibited a normal like growth rate in comparison to p110α-wt (Figure 4A, B). The presence of both early (0 to 1 h after G1 entry) and late (3 to 7 h after G1 entry) waves of PI3K activity is thought to be necessary for the induction of DNA synthesis and cell cycle progression (Jones and Kazlauskas 2001). Our results implicate that geminin degron mediated p110α degradation in late M-early G1 is more severely impairing cell cycle progression than its later degradation induced by Cdt1 degron. We verified these results by conducting cell viability assays with p110α-Gem and p110α-Cdt1 expressing DKO MEFs using CellTiter-Glo, which detects cell numbers indirectly by measuring ATP production in metabolically active cells. Our results again imply that p110α-Gem expression poorly compensated for endogenous p110α, as p110α-Cdt1 expressing DKO MEFs grew twice as fast as the p110α-Gem expressing ones (Figure 4C). Then, we set to analyze cell cycle profiles of exponentially growing add-back MEFs with FACS analysis (Figure 4D). p110α-wt expressing DKO MEFs have a relatively high mitotic index (G2-M population = 28%), in comparison to Gem and Cdt1 degron p110α expressing MEFs (18% and 21%, respectively, Figure 4E). Interestingly, although p110α-Cdt1 expression had only a minor effect on the S phase population in DKO MEFs, p110α-Gem expressing DKO MEFs had a substantially smaller S-phase population (9% vs 19%) as well as a larger G1 population. Collectively, these results indicate that the reduced proliferative potential of p110α-Gem expressing DKO MEFs is associated with a prolonged G1 as well as a reduced proportion of S-phase cells. On the other hand, S-phase specific degradation of p110α through Cdt1 degron did not diminish the proliferation of MEFs. It seems, as though, the late M/early G1 function of p110α, probably in the form of PIP_3_ generation, is more critical than its role as a source of PIP_3_, later on in the cell cycle.

### 3.3. p110α activity at the M-to-G1 transition is critical for reentry into the cell cycle upon serum withdrawal

**Figure 5:**
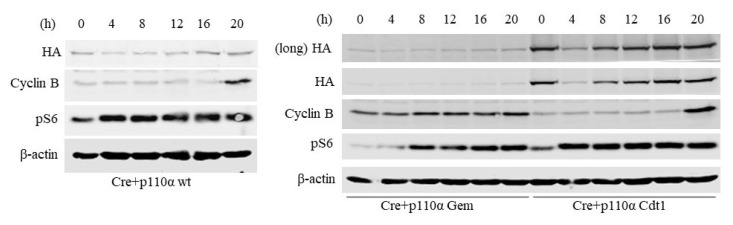
Early-G1 specific p110α activity is indispensable for promotion of cell cycle reentry upon serum stimulation. p110α-wt, p110α-Gem, and p110α-Cdt1 expressing DKO MEFs were serum starved for 24 h and were allowed to reenter into the cell cycle upon stimulation with 8% FBS for indicated time intervals. Anti HA antibodies detected expression of ectopic p110α, whereas anti cyclin B serves as a marker for M phase. PI3K signaling efficiency was determined with p-S6 immunoblots. Anti β-actin blots serve as loading control for all panels.

The choice to cross the R point is the most important step in regular control of cellular proliferation. (Lundberg and Weinberg 1998). Subsequently, we aimed to determine whether late M/early G1 degredation of p110α compromise the transition from quiescence induced by serum starvation into proliferative state. Untransformed cells lose their ability to proliferate in the absence of growth factors and exit the cell cycle. Hence, we serum starved MEFs for 24 h to induce a cell cycle block. Subsequent to serum starvation, cells were fed normal growth medium containing serum (8% FBS) and collected at regular time intervals up to 20 h. Cell cycle reentry was traced by examining levels of cyclin B, which is a critical component of Cdk1 complex triggering G2-M phase transition. In p110α-wt expressing control cells, accumulation of cyclin B occurs 20 h post serum stimulation, which is also accompanied by an increase in p-S6 consistent with gradual activation of PI3K pathway. Interestingly, p110α-Cdt1 expressing DKO MEFs, but not p110α-Gem DKO MEFs, exhibited a similar pattern of protein abundance for cyclin B (Figure 5). These results indicate that presence of p110α in M-G1 phases is crucial not only for cell cycle transitions but also for reentry into the cell cycle upon subsequent serum starvation and stimulation.

## 4. Discussion

PI3K signaling pathway initiates a series of tightly regulated biochemical processes at the plasma membrane, which are required for cell cycle entry and progression. Several isoforms of lipid phosphorylating PI3Ks exist in eukaryotic cells and relative contributions of these different PI3K isoforms to cell cycle progression as well as their activation patterns in distinct stages of the cell cycle remains obscure. Here, we have used a molecular genetic cellular model that enabled us to express the essential *PIK3CA* gene (encoding p110α) in distinct stages of the cell cycle, making use of differentially regulated degron sequences. In terms of cellular proliferation and cell cycle reentry, this technique allowed us to make functional comparisons between wild type and cell cycle-controlled variants of *PIK3CA*. By knocking out endogeous PI3Ks, we abrogated functional compensation and were able to focus on functionality of the ectopic p110α variants.

Our degron tagged *PIK3CA* constructs were well behaved when tested under the guidance of existing literature. They were responsive to serum stimulation by upregulating phospho-Akt and S6 on PI3K target phosphorylation sites and regulated p110 abundance in a way amenable to the previous reports; namely, ‘Geminin degron’ enabled a decrease in protein levels in M and early G1, whereas ‘Cdt1 degron’ promoted S-phase destabilization of the tagged p110α. Furthermore, our efforts in selectively targeting p110α in early G1 and S phases, respectively were successful as judged by several means of cell synchronization methods, e.g., nocodozole treatment, by disturbing microtubule network allowed M phase enrichment, excess thymidine exposure induced an S-phase blockage by saturating ribonucleotide reductase, whereas serum withdrawal promoted a reversible cell cycle exit. Persistence of minimal degron tagged p110α constructs in each case might be attributed to the stabilizing effect of interactions with the regulatory p85 subunit, mediated via its SH2 and/or SH3 domains.

In conclusion, our findings reveal a novel contrast between p110α’s early G1 and S-phase roles and shed new light on how PI3K activation regulates the cell cycle. Besides controlling early stages of the cell cycle, PI3K has been shown to modulate G2 and M phases as well. Here, we propose that lipid kinase p110α activity in early G1 is essential for cell cycle transitions, and its secondary activation during S-phase is dispensible for progression through the cell cycle. Collectively, our results highlight the importance of the early wave of p110α activity in controlling cellular proliferation.

Solid tumors are heterogenous masses of transformed cells. A simple mitotic staining or FACS profiling of a tumor sample could in principle determine their relative cell cycle distribution. Based on our data, tumor cells enriched in M and G1 phases of the cell cycle might be more vulnerable to PI3K targetted therapies, e.g., alpelisib (BYL-719), in HR+, HER2−, and PIK3CA mutated advanced breast cancers (Narayan et al.,, 2021). A similar degron tag study could be employed to elucidate cell cycle specific control imposed by p110β, which is known to have lipid kinase independent roles in the nucleus (Kumar et al., 2011). Our data warrant further research into the role of the other class IA PI3K isoforms in regulation of cell cycle transitions and offer novel therapeutic approaches concerning treatment of PI3K activated cancers.
